# *ABCB1* Gene Is Associated With Clinical Response to SNRIs in a Local Chinese Han Population

**DOI:** 10.3389/fphar.2019.00761

**Published:** 2019-07-04

**Authors:** Xiao-Xiao Shan, Yan Qiu, Wei-Wei Xie, Ren-Rong Wu, Yan Yu, Hai-Shan Wu, Le-Hua Li

**Affiliations:** ^1^Department of Psychiatry, the Second Xiangya Hospital, Central South University, Changsha, China; ^2^Mental Health Institute of the Second Xiangya Hospital, Central South University, Chinese National Clinical Research Center on Mental Disorders, Chinese National Technology Institute on Mental Disorders, Human Key Laboratory of Psychiatry and Mental Health, Changsha, China; ^3^Department of Psychiatry, Ningbo Kangning Hospital, Ningbo, China; ^4^The People’s Hospital of Hunan Province, Changsha, China

**Keywords:** ABCB1 gene, clinical response, SNRIs, major depressive disorder, local Chinese Han population

## Abstract

**Background:** The relation between the *ATP-binding cassette subfamily B member 1* (*ABCB1*) gene and major depressive disorder (MDD) has been studied in a local Chinese Han population. MDD is associated with the rs2032582 (*G2677T*) and rs1128503 (*C1236T*) single-nucleotide polymorphisms (SNPs) of *ABCB1* but not with rs1045642, rs2032583, rs2235040, and rs2235015. This study aims to explore the potential correlations of therapeutic responses with selective serotonin reuptake inhibitors (SSRIs) and serotonin-norepinephrine reuptake inhibitors (SNRIs) in a local Chinese Han population.

**Methods:** The study population included 292 patients with MDD. All patients were assessed at baseline and at first, second, fourth, and sixth weeks according to the 17-item Hamilton Rating Scale for Depression (HAM-D17) to determine their therapeutic responses to SSRIs and SNRIs.

**Results:** In the SSRI therapy group, the genotype or allele distribution of six SNPs was not significantly different between responders and nonresponders. In the SNRI therapy group, only rs2032583 was associated with a therapeutic response to SNRIs. The C allele of the *ABCB1* rs2032583 polymorphism was negatively correlated with therapeutic responses according to logistic regression analysis.

**Conclusion:** The *ABCB1 gene polymorphisms may* not be associated with therapeutic responses to SSRIs but not with SNRIs. The TT genotype of rs2032583 could be a predictive factor of improved treatment responses to SNRIs in the Chinese population. These findings should be replicated in future studies with larger patient groups.

## Introduction

The *ATP-binding cassette subfamily B member 1* (*ABCB1*) gene, a *multidrug resistance protein 1* (*MDR1*) gene, is located on the chromosomal region 7q21.1 and encodes p-glycoprotein (P-gp), which plays an important role in drug bioavailability and response to drugs. P-gp is a 1280-amino acid transporter and serves as a genetically polymorphic efflux transporter that removes foreign substances from cells. This protein is expressed in the blood–brain barrier and protects the brain from drugs or neurotoxic substances, such as glucocorticoids and amyloid beta (de Klerk et al., 2013).

After treatment with regular doses of antidepressants, several patients with major depressive disorder (MDD) fail to obtain satisfactory therapeutic effects, and some patients even incur serious side effects. Among patients with MDD treated with a single antidepressant medication, only 50% of patients received adequate clinical response, and 30% of patients achieved recovery. There was a delayed response to symptom relief, ranging from 4 to 8 weeks. During this period, the risk of suicide increased significantly, whereas another 10% of the patients were ineffective for any kind of antidepressant medication (Weizman et al., 2012). *ABCB1* gene polymorphisms affect the ability of drugs to pass through the blood–brain barrier into the central nervous system, leading to inadequate drug concentrations in the brain (Uhr et al., 2008). *ABCB1* gene polymorphisms can predict the effect of antidepressant drugs that are *MDR1* substrates (Mihaljevic Peles et al., 2008; Sarginson et al., 2010).

Some drugs have been identified as substrates of P-gp. These drugs include nortriptyline, imipramine, escitalopram, amitriptyline, paroxetine, venlafaxine, and citalopram; and those with non-*ABCB1* substrates include bupropion, mirtazapine, and fluoxetine (Mihaljevic Peles et al., 2008). The *ABCB1* gene has single-nucleotide polymorphisms (SNPs) in the encoding regions. Variants such as C3435T (rs1045642), G2677T/A (rs2032582), and rs2032583 are the most commonly studied. Ameyaw et al. (2001) reported that *ABCB1* gene knockout mice possessed insufficient P-gp, leading to high drug concentrations in the blood and weak ability to eliminate drugs. The plasma concentrations of drugs in mice without the *ABCB1* gene were fivefold higher and seven- to 36-fold higher in the cerebrospinal fluid (Ameyaw et al., 2001).The concentrations of citalopram, venlafaxine, and d-venlafaxine in the brain of mutant mice were 3.0, 1.7, and 4.1 times higher than those in their wild-type littermates (Uhr et al., 2008). *In vivo* studies indicated that mdr1 ab (−/−) mutant mice possessed higher cerebral concentrations of paroxetine compared with those of mdr1ab (+/+) control mice. This finding suggests that P-gp could prevent paroxetine from entering into the brain (Uhr et al., 2003). Patients with the CC genotype of SNP rs2232583 in the *ABCB1* gene exhibited a higher response rate than that of patients with the TT genotype after 4 weeks of antidepressant treatment. However, in the non-P-gp substrate mirtazapine group, no difference in genotype was observed between remission and nonremission groups (Uhr et al., 2007). A similar result was also found by Sarginson et al. (2010). Moreover, Dong et al. reported that *ABCB1* gene polymorphisms rs4728697, rs2032583, and rs58898486 were associated with depression therapeutic response, and rs17064 was related to the curative effect of desipramine (Dong et al., 2009). *ABCB1* haplotypes and SNPs rs1045642, rs2032582, and rs2032583 affect responses to antidepressant treatment (Menu et al., 2010; Rosenhagen and Uhr, 2011; Lin et al., 2011; Singh et al., 2012; de Klerk et al., 2013).

A total of 292 Chinese patients with MDD and 208 unrelated control individuals from a local Chinese Han population were studied. There are few studies on the relationship between gene polymorphism of *ABCB1* and depression in the Chinese Han population; only a team of Taiwan scholars studied the relationship between the efficacy of escitalopram and gene polymorphism of *ABCB1* in patients with depression (Lin et al., 2011). The purpose of this study was to further understand the relationship between the two in the Chinese Han population and to provide a theoretical basis for individualized treatment.

## Materials and Methods

### Subjects

A total of 292 patients with MDD and 208 healthy controls were included in this study. All participants were biologically unrelated and of Chinese Han ethnicity. The patients were diagnosed as having MDD as defined in the Axis I of the *Diagnostic and Statistical Manual of Mental Disorders, Fourth Edition, Text Revision* (DSM-IV-TR) and obtained scores ≥18 by the 17-item Hamilton Depression (HAM-D17) Rating Scale. A consensus diagnosis by at least two psychiatrists was made for each patient according to the DSM-IV criteria. Patients were not eligible to participate in the study if they have any other mental disorder according to DSM-IV-TR Axis I criteria, had major physical and neurological illnesses and sequelae of serious illness, or serious suicide attempts and behavior. Patients were also excluded if they had used electroconvulsive therapy or antipsychotic drugs with long-lasting effects within the last 6 months or any antipsychotic drugs within the last 4 weeks. The mean ages ± standard deviations of the patients and controls were 30.89 ± 10.92 years and 31.71 ± 8.25 years, respectively. Among the 292 patients with MDD, 71.6% had a single episode and 28.4% had recurrent episodes. The study protocol was approved by the Medical Ethics Committee of Second Xiangya Hospital, Central South University. Written informed consent was obtained from each patient after the study was explained.

### Study Design

Eligible patients were treated with one of the five antidepressants (escitalopram, paroxetine, sertraline, duloxetine, and venlafaxine) for 6 weeks. All patients affirmed a regular dose intake of antidepressant drug per day during the study. The primary efficacy measurement was the change in the HAM-D17 total score from baseline until the end of the study period. Patients were evaluated at screening, baseline, and on the first, second, fourth, and sixth weeks of treatment. Response was defined as changes in the HAM-D17 total score of ≥50%.

### Sample Collection and DNA Extraction

Peripheral blood samples were collected from ethylene diamine tetraacetic acid (EDTA)-containing tubes following the standard venipuncture technique. Genomic DNA was extracted from whole blood according to standard procedures. In this study, we selected SNPs by the following three methods: literature reviewing, searching for Tag-SNP, and searching for functional variant sites by FAST SNP. Finally, we investigated the following six SNPs of the *ABCB1* gene: SNP1 (rs1045642) in exon 27, SNP2 (rs2032583) in intron 22, SNP3 (rs2032582) in exon 22, SNP4 (rs2235040) in intron boundary exon 21, SNP5 (rs1128503) in exon 13, and SNP6 (rs2235015) in intron 5 of the *ABCB1* gene. All genotyping experiments were carried out by Shanghai BioWing Applied Biotechnology Company (http://www.biowing.com.cn). The AxyPrep Blood Genomic DNA Kit was used for extraction, and the ligase detection reaction (LDR) was used to detect the six SNPs. The LDR was performed in 30 cycles at 95°C for 2 min, 94°C for 15 s, and 50°C for 25 s. Target DNA sequences were amplified using a multiplex polymerase chain reaction method. The fluorescent products of the LDR were differentiated using a 3730 ABI sequencer.

### Haplotype and Statistical Analysis

Two independent sample *t*-test and χ^2^ test were used to examine the clinical and demographic variables between responders and nonresponders. Genotype and allele frequency distributions were compared between the patients and controls and between the responders and nonresponders using the χ^2^ test for independence. The observed genotype frequencies were compared with the predicted frequencies to investigate the concordance with the Hardy–Weinberg (H-W) equilibrium. Logistic regression analysis was used to estimate the therapeutic effect associated with each genotype; odds ratios with 95% confidence intervals were obtained. A *p* < 0.05 was considered to be statistically significant. The SHEsis online analysis software was used for linkage disequilibrium and haplotype analysis. Logistic regression analyses were performed using Statistical Package for the Social Sciences version 17.0 for Windows software (SPSS Inc., Chicago, IL). Adjustment for multiple comparisons was performed by Bonferroni correction.

## Results

### Comparison of General Data Between Control and Case Groups

A total of 208 cases in the control group, 101 males and 107 females, the average age was 31.71 ± 8.25 years. There were 292 patients in the study group, 143 males and 149 females; the average age was 30.89 ± 10.92 years. There was no significant difference in gender and age composition between the control group and the case group (χ^2^ = 0.008, *p* = 0.927; *t* = 0.954, *p* = 0.341), which was comparable.

### Hardy–Weinberg Balance Analysis of *ABCB1* Gene Polymorphisms in the Control and Case Groups

Among the 208 control subjects, the theoretical number of genotypes was 81, the number of genotypes of CT was 98, and the number of genotypes of TT was 29. H-W analysis showed that there was no significant difference in the actual genotype distribution of rsl045642 SNP and the theoretical gene type distribution under H-W equilibrium, χ^2^ = 1.231, *p* = 0.267. According to this method, the H-W balance test was performed on the polymorphic loci rsl045642, rs2032583, rs2032582, rs2235040, rsl128503, and rs2235015 between the control group and the case group. As shown in **Table 1**, the six SNPs loci in the control and case groups all met the H-W balance. The population selected in this study is representative of the Han population and suitable for genetic analysis.

**Table 1:** Equilibrium test of six single-nucleotide polymorphisms (SNPs) between the control group and the case group.

SNP	Genotype	Controls (*n* = 208)				Cases (*n* = 292)			
		Actual number	Theoretical number	**χ** ^2^	p	Actual number	Theoretical number	**χ** ^2^	p
rs1045642	CC	85	81	1.23	0.267	103	109	2.27	0.132
	CT	90	98			151	139		
	TT	33	29			38	44		
rs2032583	CT	22	21	0.65	0.42	38	37	1.41	0.234
	TT	186	187			254	255		
rs2032582	GG	78	72	3.39	0.065	86	78	3.44	0.06
	GT	88	101			130	146		
	TT	42	36			76	68		
rs2235040	AG	22	21	0.65	0.42	40	38	1.58	0.21
	GG	186	187			252	254		
rs1128503	CC	39	34	1.8	0.18	40	35	1.97	0.161
	CT	91	100			121	132		
	TT	78	73			131	126		
rs2235015	GG	187	188	0.59	0.44	254	255	1.414	0.23
	GT	21	20			38	37		

### Comparison of Clinical Features Among the Responders and Nonresponders

Among the 292 patients included in this study, 39 patients dropped out because of adverse effects (*n* = 12), withdrawal of consent (*n* = 9), contrary to the scheme (*n* = 2), and lost to follow-up (*n* = 16); lastly, 253 patients completed the study. Clinical characteristics, use of drugs, and average drug doses between responders and nonresponders are shown in **Table 2**. No significant differences were found between the two groups according to the above indicators.

**Table 2:** Clinical features, dosage, and drug among the responders and the nonresponders.

		Nonresponders (*n* = 54)	Responders (*n* = 199)	F	*p*
Age (years)		31.19 ± 10.124	30.78 ± 11.484	1.089	0.298
Weight		60.778 ± 9.162	57.31 ± 9.3811	0.033	0.856
Education		12.17 ± 3.994	11.93 ± 3.968	0.274	0.601
Sex	Male *n* (%)	27(50)	98(49.2)	0.012	0.912
	Female *n* (%)	27(50)	101(50.8)		
Marriage	Spinsterhood *n* (%)	29(53.7)	105(52.8)	2.441	0.119
	Married *n* (%)	21(38.9)	86(43.2)		
	Divorced *n* (%)	3(5.6)	8(4.0)		
	Remarriage *n* (%)	1(1.9)	0(0.0)		
Drug	Escitalopram *n* (%)	19(35.2)	67(33.7)	5.713	
	Paroxetine *n* (%)	6(11.1)	44(22.1)		
	Venlafaxine *n* (%)	19(35.2)	46(23.1)		0.222
	Duloxetine *n* (%)	7(13.0)	34(17.1)		
	Sertraline *n* (%)	3(5.6)	8(4.0)		
Doses	Escitalopram	10.79 ± 2.507	10.67 ± 2.446	3.723	
	Paroxetine	21.67 ± 4.082	20.91 ± 2.908		
	Venlafaxine	157.89 ± 23.648	158.15 ± 23.602		0.055
	Duloxetine	60.0 ± 0.00	60.0 ± 0.00		
	Sertraline	58.33 ± 14.434	105.63 ± 52.470		

### Genotype and Allele Frequencies in the Responders and Nonresponders

Genotype and allele distributions for the examined *ABCB1* gene SNPs in nonresponders and responders are shown in **Table 3**. The genotype and allelic distributions of rs1045642, rs2032582, rs2235040, rs1128503, and rs2235015 SNPs were not significantly different between nonresponders and responders. For rs2032583, genotype and allelic distributions significantly differed between the nonresponders and responders. The distribution of TT genotype and T allele frequency was higher in the responders than that in the nonresponders (*p* = 0.027, *p* = 0.033, respectively).

**Table 3:** Genotype and allele frequencies of six SNPs of the ABCB1 gene in the nonresponders and the responders.

Genotype/allele	Responders (n = 199)(%)	Nonresponders (n = 54)(%)	**χ** ^2^	*p*	OR (95% CI)		Adjust OR (95% CI)	
rs1045642								
CC	73 (36.7)	21 (38.9)			1		1	
CT	97 (48.7)	26 (48.1)	0.136	0.934	1.073	0.560–2.056	1.159	0.575–2.336
TT	29 (14.6)	7 (13.0)			1.192	0.457–3.105	0.826	0.301–2.270
C allele	243 (61.1)	68 (63.0)			1		1	
T allele	155 (38.9)	40 (37.0)	0.131	0.718	1.084	0.699–1.683	0.762	0.299–1.945
rs2032583								
TT	175 (87.9)	41 (75.9)			1		1	
CT	24 (12.1)	13 (24.1)	4.91	0.027*	0.433*	0.203–0.921	0.4*	0.179–0.896
T allele	374 (94.0)	95 (88.0)			1		1	
C allele	24 (6.0)	13 (12.0)	4.52	0.033*	0.469*	0.23–0.955	0.4*	0.179–0.8966
rs2032582								
GG	63 (31.7)	16 (29.6)			1		1	
GT	81 (40.7)	25 (46.3)	0.574	0.751	0.823	0.405–1.671	0.878	0.41–1.877
TT	55 (27.6)	13 (24.1)			1.074	0.475–2.431	0.917	0.386–2.178
G allele	207 (52.0)	57 (52.8)			1		1	
T allele	191 (48.0)	51 (47.2)	0.02	0.887	1.031	0.674–1.579	0.989	0.473–2.07
rs2235040								
AG	27 (13.6)	12 (22.2)			1		1	
GG	172 (86.4)	42 (77.8)	2.44	0.118	1.82	0.852–3.888	2.031	0.905–4.558
A allele	27 (6.8)	12 (11.1)			1		1	
G allele	371 (93.2)	96 (88.9)	2.236	0.135	1.718	0.839–3.515	2.047	0.919–4.56
rs1128503								
CC	26 (13.1)	8 (14.8)			1		1	
CT	79 (39.7)	27 (50.0)	2.554	0.279	0.9	0.364–2.225	0.905	0.348–2.352
C allele	131 (32.9)	43 (39.8)			1		1	
T allele	267 (67.1)	65 (60.2)	1.793	0.181	1.348	0.87–2.09	1.41	0.727–2.734
rs2235015								
GG	174 (87.4)	42 (77.8)			1		1	
GT	25 (12.6)	12 (22.2)	3.174	0.075	0.503	0.234–1.082	0.416	0.204–1.04
G allele	373 (93.7)	96 (88.9)			1		1	
T allele	25 (6.3)	12 (11.1)	2.924	0.087	0.536	0.26–1.108	0.530	0.21–1.01

*Statistically significant difference between the groups (p < 0.05).

CI, confidence interval; OR, odds ratio.

### *ABCB1* Gene Polymorphism Loci and Clinical Response to Selective Serotonin Reuptake Inhibitors

For SSRIs (sertraline, paroxetine, and escitalopram), no significant difference in genotype and allele frequency distribution was observed between the responders and nonresponders (*p* > 0.05) (**Table 4**).

**Table 4:** Genotype, allelic distribution of all genotyped single nucleotide polymorphisms among the treatment responders and nonresponders for selective serotonin reuptake inhibitor (SSRI) drugs group.

	Responders (*n* = 119) (%)	Nonresponders (*n* = 28) (%)	χ^2^	*p*	OR (95% CI)	Adjust OR (95% CI)
rs1045642						
CC	42 (35.3)	9 (32.1)			1	1
CT	60 (50.4)	14 (50.0)	0.259	0.879	0.918 (0.364–2.317)	0.717 (0.255–2.02)
TT	17 (14.3)	5 (17.9)			0.729 (0.213–2.492)	0.389 (0.098–1.551)
C allele	144 (60.5)	32 (57.1)			1	1
T allele	94 (39.5)	24 (42.9)	0.213	0.644	0.87 (0.483–1.57)	0.479 (0.143–1.601)
rs2032583					
TT	106 (89.1)	24 (85.7)			1	1
CT	13 (10.9)	4 (14.3)	0.25	0.617	0.736 (0.221–2.455)	0.751 (0.205–2.747)
T allele	225 (94.5)	52 (92.9)			1	1
C allele	13 (5.5)	4 (7.1)	0.235	0.628	0.751 (0.235–2.397)	0.751 (0.205–2.747)
rs2032582					
GG	38 (31.9)	5 (17.9)			1	1
GT	53 (44.5)	14 (50.0)	2.352	0.309	0.498 (0.165–1.501)	0.336 (0.09–1.256)
TT	28 (23.5)	9 (32.1)			0.409 (0.124–1.355)	0.237 (0.057–0.984)
G allele	129 (54.2)	24 (42.9)			1	1
T allele	109 (45.8)	32 (57.1)	2.338	0.126	0.634 (0.352–1.14)	0.509 (0.189–1.37)
rs2235040					
AG	15 (12.6)	4 (14.3)			1	1
GG	104 (87.4)	24 (85.7)	0.056	0.813	1.156 (0.352–3.794)	1.129 (0.316–4.032)
A allele	15 (6.3)	4 (7.1)			1	1
G allele	223 (93.7)	52 (92.9)	0.052	0.82	1.144 (0.364–3.588)	1.129 (0.316–4.032)
rs1128503					
CC	16 (13.4)	2 (7.1)			1	1
CT	39 (32.8)	14 (50.0)	3.124	0.21	0.348 (0.071–1.711)	0.303 (0.057–1.627)
TT	64 (53.8)	12 (42.9)			0.667 (0.135–3.282)	0.483 (0.091–2.57)
C allele	71 (29.8)	18 (32.1)			1	1
T allele	167 (70.2)	38 (67.9)	0.115	0.735	1.114 (0.596–2.083)	1.224 (0.494–3.033)
rs2235015					
GG	106 (89.1)	24 (85.7)			1	1
GT	13 (10.9)	4 (14.3)	0.25	0.617	0.736 (0.221–2.455)	0.696 (0.191–2.543)
G allele	225 (94.6)	52 (92.9)			1	1
T allele	13 (5.5)	4 (7.1)	0.235	0.628	0.751 (0.235–2.397)	0.751 (0.235–2.397)

**Figure 1:**
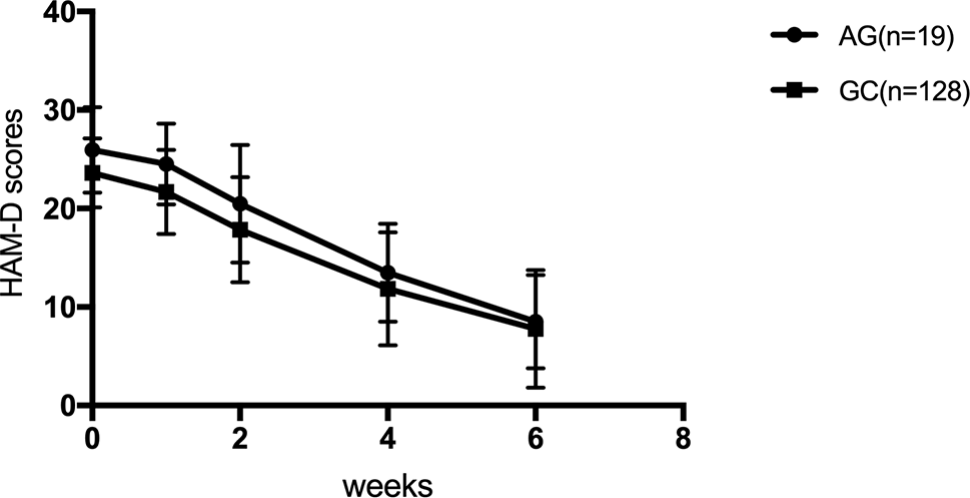
Hamilton Rating Scale for Depression (HAM-D) scores at the first, second, fourth, and sixth week.

The efficacy of SSRIs was examined for genotypes in the SSRI treatment group according to the HAM-D17 scores, decreased scores, and reducing score rate during the first, second, fourth, and sixth weeks. The HAM-D17 scores, decreased scores, and reducing score rate at the first, second, fourth, and sixth weeks were not significantly different among rs1045642, rs2032583, rs2032582, rs1128503, and rs2235015 (*p* > 0.05). HAM-D17 decreased scores and reducing score rate did not reveal any significant difference with rs2235040 (**Table 5**); however, a significant difference was observed for the genotype of rs2235040 in the HAM-D17 scores (F = 4.349, *p* = 0.039) (**Figure 1**).

**Table 5:** Rs2235040 and response to antidepressants.

		1 week	2 weeks	4 weeks	6 weeks	F	p
Decreased score	AG (*n* = 19)	1.42 ± 1.71	5.47 ± 5.28	12.47 ± 5.78	17.42 ± 5.27	0.147	0.702
	GG (*n* = 128)	1.93 ± 2.49	5.74 ± 4.57	11.75 ± 5.99	15.82 ± 6.37		
Reducing score rate (%)	AG (*n* = 19)	5.31 ± 6.43	20.74 ± 18.96	47.36 ± 19.63	67.3 ± 17.42	0.323	0.571
	GG (*n* = 128)	8.38 ± 11.31	24.50 ± 19.09	49.64 ± 22.97	67.04 ± 23.77		

### *ABCB1* Gene Polymorphism Loci and Clinical Response to Serotonin–Norepinephrine Reuptake Inhibitors

For SNRIs (venlafaxine and duloxetine), no significant difference was observed in the distribution of genotype and allele frequency of the rs1045642, rs2032582, rs2235040, rs1128503, and rs2235015 SNPs between the responders and nonresponders (*p* > 0.05). For rs2032583, the T allele frequency and TT genotype were significantly increased in the responders compared with those in the nonresponders (*p* = 0.025 and *p* = 0.018, respectively) (**Table 6**).

**Table 6:** Genotype, allelic distribution of all genotyped SNPs among the treatment responders and nonresponders for serotonin-norepinephrine reuptake inhibitors (SNRIs).

	Responders (*n* = 80) (%)	Nonresponders (*n* = 26) (%)	X^2^	*p*	OR (95% CI)	Adjust OR (95% CI)
rs1045642						
CC	31 (38.8)	12 (46.2)			1	1
CT	37 (46.3)	12 (46.2)	1.059	0.589	1.194 (0.47–3.03)	1.898 (0.644–5.593)
TT	12 (15.0)	2 (7.7)			2.323 (0.451–11.96)	1.499 (0.263–8.537)
C allele	99 (61.9)	36 (69.2)			1	1
T allele	61 (38.1)	16 (30.8)	0.918	0.338	1.386 (0.71–2.709)	1.11 (0.208–5.932)
rS2032583						
TT	69 (86.3)	17 (65.4)			1	1
CT	11 (13.8)	9 (34.6)	5.581	0.018*	0.301* (0.108–0.842)	0.261* (0.085–0.807)
T allele	149 (93.1)	43 (82.7)				1
C allele	11 (6.9)	9 (17.3)	4.999	0.025*	0.353* (0.137–0.907)	0.261* (0.085–0.807)
rs2032582						
GG	25 (31.3)	11 (42.3)			1	1
GT	28 (35)	11 (42.3)	3.254	0.196	1.12 (0.414–3.028)	1.466 (0.473–4.541)
TT	27 (33.8)	4 (15.4)			2.97 (0.836–10.55)	2.502 (0.644–9.72)
G allele	78 (48.75)	33 (63.5)			1	1
T allele	82 (51.25)	19 (36.5)	3.405	0.065	1.826 (0.959–3.477)	2.07 (0.598–7.170)
rs2235040						
AG	12 (15)	8 (30.8)			1	1
GG	68 (85)	18 (69.2)	3.188	0.074	2.519 (0.895–7.086)	2.98 (0.96–9.245)
A allele	12 (7.5)	8 (15.4)			1	1
G allele	148 (92.5)	44 (84.6)	2.856	0.091	2.242 (0.862–5.832)	2.98 (0.96–9.245)
rs1128503						
CC	10 (12.5)	6 (23.1)			1	1
CT	40 (50.0)	13 (50.0)	2.083	0.353	1.846 (0.562–6.068)	1.838 (0.495–6.818)
TT	30 (37.5)	7 (26.9)			2.571 (0.698–9.476)	1.819 (0.422–7.839)
C allele	60 (37.5)	25 (48.1)			1	1
T allele	100 (62.5)	27 (51.9)	1.828	0.176	1.543 (0.821–2.901)	1.149 (0.385–3.43)
rs2235015						
GG	68 (85.0)	18 (69.2)			1	1
GT	12 (15.0)	8 (30.8)	3.188	0.074	0.397 (0.141–1.117)	0.983 (0.944–1.024)
G allele	148 (92.5)	44 (84.6)			1	1
T allele	12 (7.5)	8 (15.4)	2.856	0.091	0.446 (0.171–1.16)	0.446 (0.171–1.16)

*Statistically significant difference between the groups (p < 0.05).

**Figure 2:**
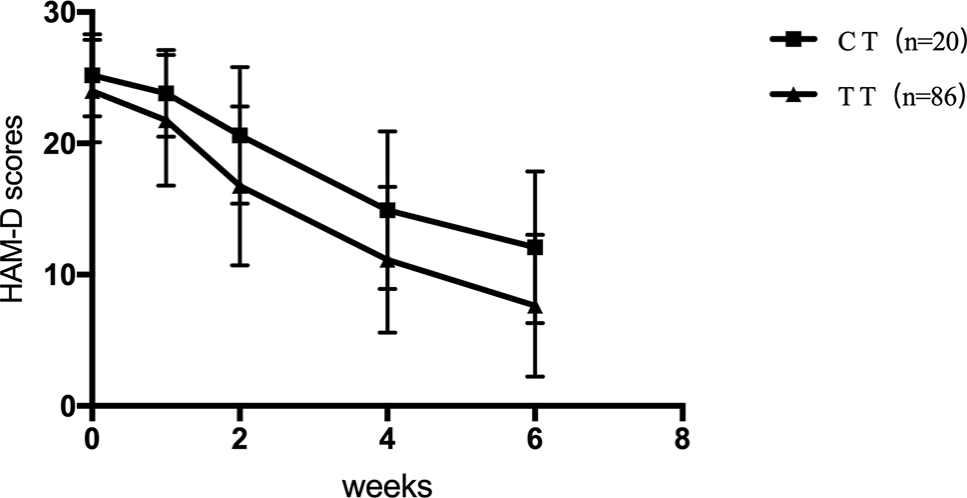
HAM-D scores at the first, second, fourth, and sixth week (Rs2032583).

**Figure 3:**
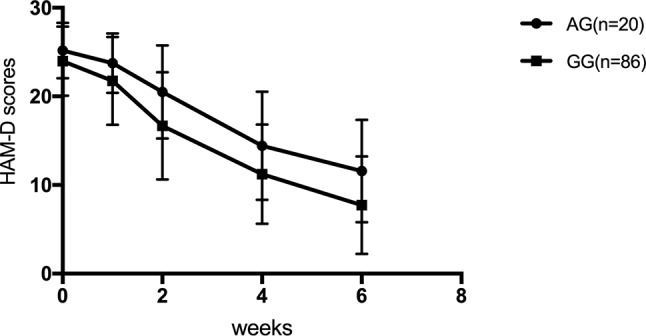
HAM-D scores at the first, second, fourth, and sixth week (Rs2235040).

**Figure 4:**
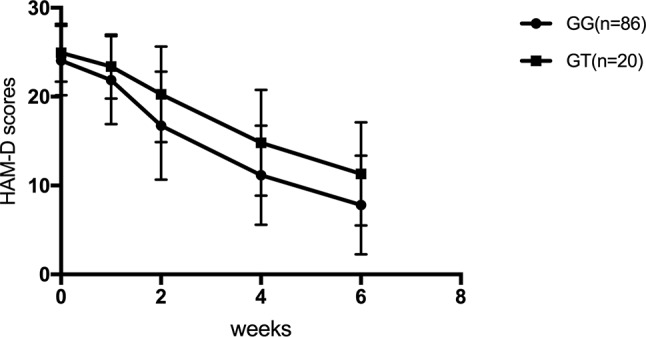
HAM-D scores at the first, second, fourth, and sixth week (Rs2235015).

For SNRIs (venlafaxine and duloxetine), no significant difference was observed for the rs1045642, rs2032582, and rs1128503 SNPs in any of the HAM-D17 scores, decreased scores, and reducing score rate during the first, second, fourth, and sixth weeks (*p* > 0.05). For rs2032583, the HAM-D17 scores of TT genotype are lower than those of the CT genotype, whereas the decreased scores and reducing score rate are higher than those of the CT genotype (**Table 7** and **Figure 2**). The GG genotypes of rs2235040 have lower HAM-D17 scores than AG genotypes and higher decreased score and reducing score rate than AG genotypes (**Table 8** and **Figure 3**). For rs2235015, the GG genotypes have lower HAM-D17 scores than those of the GT genotypes and higher in decreased score and reducing score rate than those of the GT genotypes (**Table 9** and **Figure 4**).

**Table 7:** Rs2032583 and response to antidepressants.

		1 week	2 weeks	4 weeks	6 weeks	F	*p*
Decreased score	CT (*n* = 17)	1.40 ± 1.79	4.60 ± 4.11	10.3 ± 6.33	13.1 ± 6.51	5.949	0.016*
	TT (*n* = 130)	2.23 ± 2.67	7.33 ± 4.62	12.86 ± 5.28	16.36 ± 5.29		
Reducing score rate (%)	CT (*n* = 17)	5.49 ± 7.33	18.69 ± 17.19	40.50 ± 24.09	51.4 ± 24.0	9.241	0.003*
	TT (*n* = 130)	9.71 ± 11.76	31.29 ± 19.91	53.91 ± 20.80	68.8 ± 20.77		

*Statistically significant difference between the groups (p < 0.05).

**Table 8:** Rs2235040 and response to antidepressants.

		1 week	2 weeks	4 weeks	6 weeks	F	*p*
Decreased score	AG (*n* = 20)	1.45 ± 1.76	4.70 ± 4.07	10.75 ± 6.02	13.60 ± 6.06	4.288	0.041*
	GG (*n* = 86)	2.22 ± 2.68	7.30 ± 4.64	12.76 ± 5.41	16.24 ± 5.47		
Reducing score rate (%)	AG (*n* = 20)	5.74 ± 7.21	19.19 ± 17.07	42.75 ± 23.17	53.9 ± 22.57	6.64	0.011*
	GG (*n* = 86)	9.65 ± 11.80	31.18 ± 20.00	53.39 ± 21.33	68.22 ± 21.56		

*Statistically significant difference between the groups (p < 0.05).

**Table 9:** Rs2235015 and response to antidepressants.

		1 week	2 weeks	4 weeks	6 weeks	F	*p*
Decreased score	GG (*n* = 130)	2.20 ± 2.67	7.30 ± 4.64	12.90 ± 5.28	16.23 ± 5.42	4.93	0.029*
	GT (*n* = 17)	1.55 ± 1.85	4.70 ± 4.08	10.15 ± 6.25	13.65 ± 6.29		
Reducing score rate (%)	GG (*n* = 130)	9.53 ± 11.75	31.12 ± 20.0	53.96 ± 20.86	68.1 ± 21.32	7.151	0.009*
	GT (*n* = 17)	6.27 ± 7.82	19.44 ± 17.28	40.31 ± 23.73	54.4 ± 23.90		

*Statistically significant difference between the groups (p < 0.05).

## Discussion

We investigated the association among the six SNPs of the *ABCB1* gene and therapeutic response in the local Chinese Han population. Among the five SNPs, only one SNP (rs2032583) differed in genotype and allele frequencies between the responders and nonresponders. In particular, the TT genotype of this SNP was significantly more common in the responders than that in the nonresponders. This finding suggests that the C allele of rs2032583 may be a risk factor for MDD. In the SSRI therapy group, no correlation was found between the six SNPs of the *ABCB1*gene and therapeutic response to SSRIs (*p* > 0.05). However, for the SNRI therapy group, only rs2032583 is associated with a therapeutic response to SNRIs in patients with MDD. The C allele of the *ABCB 1* gene SNP rs2032583 was negatively correlated with therapeutic response according to the logistic regression analyses. The genotype of *ABCB1* gene SNPs rs2235015 and rs2235040 were associated with decreased score and reducing score rate in the SNRIs therapy group, but because of the small sample size, there was no significant difference in the final effective and ineffective grouping. This finding indicated that *ABCB1* gene polymorphisms may not be associated with the treatment response to SSRIs, but with SNRIs. The TT genotype of the *ABCB1* gene SNP rs2032583 could be a predictive factor of improved treatment response to SNRIs. To our knowledge, this study is the first to report the genetic association of *ABCB1* gene polymorphism with therapeutic responses in a case-control design in Chinese Han people in Mainland China.

P-gp can restrict the entry of various substrates, such as antidepressants, from the bloodstream into the brain. Studies on *ABCB1* knockout animals have shown that *ABCB1*−/− mice possess higher intracerebral concentrations of escitalopram, trimipramine, amitriptyline, doxepin, venlafaxine, and paroxetine compared with those in wild-type mice (Uhr et al., 2000; Uhr et al., 2002; Uhr et al., 2003). Kato et al. (2008) reported a significant association of the nonsynonymous SNP *G2677T/A* (rs2032582) with treatment response to paroxetine in depressed patients. Moreover, the wild variant haplotype *3435C–2677G–1236T* is associated with poor response (Kato et al., 2008). In contrast, Nikisch et al. (2008) found that depressed patients harboring the *2677 GG/GT* genotype responded to escitalopram treatment significantly better than patients with the *2677TT* genotype and suggested that this polymorphism could be used as genetic markers for predicting treatment response to escitalopram treatment in MDD (*n* = 15). Uhr et al. (2008) showed that polymorphisms in the *ABCB1* gene predict the response to antidepressant treatment in depressed patients (*n* = 133) receiving drugs that have been identified as substrates of P-gp (amitriptyline, paroxetine, venlafaxine, and escitalopram). However, Mihaljevic et al. (2008) found that MDR1 variants *G2677T* (rs2032582) and *C3435T* (rs1045642) are not associated with therapeutic response to paroxetine in patients with MDD (*n* = 127). Furthermore, Peters et al. (2008) did not find an association between *ABCB1C3435T*, *G2677T*, and *C1236T* polymorphisms and response and tolerance to citalopram in a large study sample (*n* = 831).

*ABCB1* gene polymorphism loci mutation might cause the variation of P-gp, thereby increasing drug concentrations in the brain to improve therapeutic effects. The specific function of rs2032583 (intron 22) is not clear. Clinical research has found the association between rs2032583 and antidepressant effects. The wild-type allele of rs2032583 is T and the mutant allele is C. The study by Sarginson et al. (2010) showed that carriers of the C allele remitted faster than those with the TT genotype in the rs2032583 among paroxetine-treated patients and support the findings of Uhr et al. (2002) who found that rs2032583 genetic variants affected efficacy in patients of various ages treated with paroxetine and other *ABCB1* substrates (Uhr et al., 2008; Sarginson et al., 2010). By contrast, the two previous results are inconsistent with our present results. For rs2032583, we found that TT genotype has lower HAM-D17 scores than those of CT genotype and higher in decreased score and reducing score rate than those of CT genotype. This finding suggested that the TT genotype of rs2032583 is likely to be a predictive factor of better treatment response to SNRIs.

Whether the locus mutation manipulates the coding region of P-protein conformation remains to be further basic research. The rs2032583 locus mutation might change the dimensional conformation of P-gp by allowing entry of drugs and toxic substances into the brain simultaneously to exacerbate the symptoms. In addition, the locus mutation changes P-gp conformation and then stops the SNRIs into the brain but does not affect SSRI substrate. Furthermore, locus mutation may not change the conformation of P-gp. The positive results of SNRIs may be attributed to the small sample size, thereby causing false-positive results in the study. Foreign studies may have different results because of various factors such as race, region, environment, and diet.

Several limitations of the present study should be acknowledged. First, we did not distinguish the first and recurrence of depressive patients joining the patients’ group. Regression analysis determines the total course of the disease and whether they accepted treatment; however, previous treatment had some influence on the overall response rate. Hence, the present study can only obtain a trend about differences. Second, the present study only selected the drugs that have been identified as substrates of P-gp. Future studies should include no-P-gp substrates such as mirtazapine. Third, genetic effect is slight because of the small sample size. A large sample size is needed to improve the power of the test. Fourth, many receptors are involved to determine the effect of antidepressant drugs; hence, a combination of gene should be considered. Larger and more homogenous samples will be included in our future research to improve the statistical power. Because there may be a delayed response to symptom relief, 6 weeks of treatment may not be sufficient to fully demonstrate the relationship between *ABCB1* gene and clinical response, and we consider extending the duration of efficacy observation in future studies.

## Ethics Statement

The study protocol was approved by the Medical Ethics Committee of Second Xiangya Hospital, Central South University. Written informed consent was obtained from each patient after the study was explained.

## Author Contributions

X-XS and YQ contributed equally to this work, mainly responsible for essay writing and conducting the study. W-WX mainly participated in conducting the study. YY assisted the study. R-RW, H-SW and L-HL contributed to guide of conducting the study and essay writing.

## Funding

This research was supported by National Natural Science Foundation of China (grant no. 81501163), National Natural Science Foundation of China (grant no. 81270019), National R&D Special Fund for Health Profession (grant no. 201002003), and National Science and Technology Major Projects for “Major New Drugs Innovation and Development” (2012ZX09303014-001).

## Conflict of Interest Statement

The authors declare that the research was conducted in the absence of any commercial or financial relationships that could be construed as a potential conflict of interest.
